# Developmental Cycle and Genome Analysis of “*Rubidus massiliensis*,” a New *Vermamoeba vermiformis* Pathogen

**DOI:** 10.3389/fcimb.2016.00031

**Published:** 2016-03-15

**Authors:** Jacques Y. Bou Khalil, Samia Benamar, Jean-Pierre Baudoin, Olivier Croce, Caroline Blanc-Tailleur, Isabelle Pagnier, Didier Raoult, Bernard La Scola

**Affiliations:** ^1^Unité de Recherche sur les Maladies Infectieuses et Tropicales Emergentes, Facultés de Médecine et de Pharmacie, UM63 Centre National de la Recherche Scientifique 7278 IRD 198 Institut National de la Santé et de la Recherche Médicale U1095, Aix-Marseille UniversitéMarseille, France; ^2^Pôle des Maladies Infectieuses et Tropicales Clinique et Biologique, Fédération de Bactériologie-Hygiène-Virologie, Centre Hospitalo-Universitaire Timone, Institut Hospitalo-Universitaire Méditerranée Infection, Assistance Publique – Hôpitaux de MarseilleMarseille, France

**Keywords:** chlamydiae, *Vermamoeba vermiformis*, co-culture, host specificity, *Rubidus massiliensis*

## Abstract

The study of amoeba-associated *Chlamydiae* is a dynamic field in which new species are increasingly reported. In the present work, we characterized the developmental cycle and analyzed the genome of a new member of this group associated with *Vermamoeba vermiformis*, we propose to name “*Rubidus massiliensis*.” This bacterium is well-adapted to its amoeba host and do not reside inside of inclusion vacuoles after phagocytosis. It has a developmental cycle typical of this family of bacteria, with a transition from condensed elementary bodies to hypodense replicative reticulate bodies. Multiplication occurs through binary fission of the reticulate bodies. The genome of “*R. massiliensis*” consists of a 2.8 Mbp chromosome and two plasmids (pRm1, pRm2) consisting of 39,075 bp and 80,897 bp, respectively, a feature that is unique within this group. The Re-analysis of the *Chlamydiales* genomes including the one of “*R. massiliensis*” slightly modified the previous phylogeny of the *tlc* gene encoding the ADP/ATP translocase. Our analysis suggested that the *tlc* gene could have been transferred to plant and algal plastids before the transfer to *Rickettsiales*, and that this gene was probably duplicated several times.

## Introduction

*Chlamydiae* and related bacteria are Gram-negative obligate intracellular bacteria that infect a wide range of hosts, including vertebrates, insects, and unicellular organisms such as free-living amoebae (Wolf et al., 1999; Longbottom and Coulter, 2003; Magnino et al., 2009). These bacteria can be pathogens or endosymbionts (Ishida et al., 2014), and they present an extreme adaptation to intracellular life manifesting in massive gene loss and a strong dependency on host-derived metabolites (Merhej et al., 2009). Some gene transfers have been observed in *Chlamydiae*. For example, the gene coding for the ATP/ADP translocase (*tlc* gene) was previously suspected to be duplicated and transferred to several other intracellular bacteria such as *Rickettsia* and *Lawsonia intracellularis* (Schmitz-Esser et al., 2004) and to plant and algal plastids (Greub and Raoult, 2003). All *Chlamydiae* possess an ATP/ADP translocase, which is a hallmark of obligate intracellular pathogens. These proteins are unique enzymes that catalyze the highly specific exchange of bacterial ADP against host ATP (Schmitz-Esser et al., 2004, 2008). All *Chlamydiae* undergo a unique intracellular developmental cycle consisting of two morphologically and physiologically different stages (Horn, 2008): the elementary body (EB) that is required to survive the extracellular environment and to infect host cells and the reticulate body (RB) that represents the intracellular replicative form. The EB generally multiplies inside a host-derived vacuole; however, in contrast to other *Chlamydiae* species, the *Neochlamydia hartmannellae* EB does not reside within a vacuole (Horn et al., 2000). High diversity of chlamydiae has been isolated by cell culture studies in a wide range of habitats. Scientific work in biosystematics and taxonomy were realized to identify and classify new Bacteria. Modern bacterial taxonomy is based on a polyphasic approach that combines phenotypic, phylogenetic and genotypic characteristics, including 16S rRNA sequence similarity. However, due to the fact that *chlamydiae* exhibit few phenotypic traits and are difficult to culture, their taxonomic affiliation is largely based on molecular, genetic and phylogenetic analyses of housekeeping genes (Greub, 2010). Pillonel et al. recommended to use nine protein sequences in order to precisely classify newly discovered isolates at the family, genus and species levels (Pillonel et al., 2015). Over the last years, following the seminal studies of Rowbotham (1980, 1998), *Acanthamoeba* sp. have been used to isolate amoeba associated microorganisms and led to the discovery of giant viruses (Pagnier et al., 2013). As a new strategy adapted in our lab for virus isolation, and in an attempt to test new protozoa to isolate new giant viruses, we adapted our co-culture method for the isolation of giant viruses from *Vermamoeba vermiformis* (Pagnier et al., 2013, 2015; Reteno et al., 2015). During the test of series of samples for virus isolation, we isolated a bacterium that belongs to a new *Chlamydiae* genus and proposed to name this isolate “*Rubidus massiliensis*” (designed as *R. massiliensis* in the manuscript). *R. massiliensis* is characterized according to pillonel et al. taxonomy method (Pillonel et al., 2015). Its development cycle and the analysis of its genome are reported herein.

## Materials and methods

### Isolation, production, developmental cycle, and quantification procedures

*R. massiliensis* was isolated from a tap water sample in our hospital (hospital la timone, Marseille-France: intensive care unit) under previously described conditions, except that we changed the strategy, and *V.vermiformis* (reference strain CD19) was used as host instead of *A. polyphaga* (La Scola et al., 2000; Pagnier et al., 2015).

The routine co-culture was prepared by inoculating *V. vermiformis* rinsed in PAS (page's amoeba saline) and suspended in modified PAS (modified PAS is the page's amoeba saline solution implemented with 18 g of glucose, 120 mg NaCl, 4 mg MgSO_4_•7H_2_O, 4 mg CaCl2•2H_2_O, 142 mg Na_2_HPO_4_•7H_2_O, 136 mg KH_2_PO_4_, 0.02 g (NH_4_)2Fe(SO_4_)_2_.6H_2_O, and 2 g of yeast extract, to prevent some amoeba's encystment) at a concentration of 10^6^ amoebas/ml) with a *R. massiliensis* suspension at a multiplicity of infection (MOI) of 10 into two 75-cm^2^ culture flasks at 30°C. The bacterial suspension of *R. massiliensis* used for the infection was produced from a single purified clone obtained by the end point dilution method. After 1 h of incubation, the amoeba monolayer was washed three times with PAS buffer to eliminate non-internalized bacteria. This time point was designated as H0. A total of 10 ml of the infected cultures were distributed into new culture flasks incubated at 30°C. A culture flask containing only amoeba was used as the negative control. At H0, H2, H4, H6, H8, H12, H18, H24, H30, H36, H42, and H48 (H corresponds to hours post infection), we prepared five slides by cyto-centrifugation of 100 μl of culture from each flask for Gram staining, Gimenez staining and DAPI nucleic acid labeling (Molecular probes, life technologies USA). Five hundred microliters were also taken for the DNA extraction, and molecular biology. The remaining 9 ml of the co-culture was centrifuged at 720 × g for 10 min, and the pellets were fixed for the transmission electron microscopy procedures.

Bacterial growth was assessed using real time PCR assays. Bacterial counts were performed using the end-point dilution method by diluting subcultures onto fresh *V. vermiformis*. The purpose is to establish the relation between bacterial concentration and cycle threshold (Ct.). For this, bacterial DNA extractions and real-time PCR were performed using 200 μL from the 500 μL of each co-culture collected at every infection time point of the cycle (H0 to H48) as previously described. The automated extraction EZ1 DNA Tissue Kit (Qiagen, Hilden, Germany) was used for this DNA extraction according to the manufacturer's instructions on a CFX96^TM^ thermocycler (BioRad Laboratories Inc.). The following primers were used: Forward: 5′-GTACTCAGGCAGTGCACTTTA-3′, Reverse: 5′-AGCGTGTGCTTAGACCAAATA-3′, and Probe: 5′-TGCTCCAATCGCTGTTGGTATCGT-3′. The amoeba quantification was performed on counting slides (kovaslides, HYCOR biomedical Inc., 90 California, USA).

### Host range

The supernatant was collected from a 1 week-old *V. vermiformis* flask infected with *R. massiliensis*, filtered through a 5 μm filter and washed three times with modified PAS medium. Then, the filtered supernatants were used for inoculation onto *A. castellanii* (strain C3) and *Dictyostelium discoideum* (strain DH1-10) (*D. discoideum*). at an MOI of 10 in a 24-well microplate seeded with 10^6^ amoebae and containing 1 ml of PAS (for *A. castellanii*) or 1 ml of modified PAS medium (for *D. discoideum*). The microplates were centrifuged at 1500 × g for 30 min and incubated for 5 days at 32°C (*A. castellanii*) or 28°C (*D. discoideum*). Amoebae were observed daily for lysis. After infection, we daily took 500 μl from the co-cultures and total DNA was extracted to estimate the number of chlamydial DNA copies using real-time quantitative PCR as described above. We tested several temperatures for the *R. massiliensis'* growth in amoebal co-culture, temperatures range was between 4 and 42°C.

### Transmission electron microscopy (TEM) and TEM tomography

The fixation and embedding procedures are realized as described in the work of Reteno et al. (2015). Electron micrographs were either obtained on a Morgagni 268D (Philips) transmission electron microscope (TEM) operated at 100 keV or on a Tecnai G20 F20 TEM (FEI) operated at 200 keV. Tomography tilt series were acquired on the G20 Cryo TEM (FEI) with the Explore 3D (FEI) software for tilt ranges of 100 or 105° with 1 or 2° increments. The mean applied defocus was −2 μm. The magnification ranged between 9600 and 29,000 with pixel sizes between 1.09 and 0.364 nm, respectively. The image size was 4096 × 4096 pixels. The average thickness of the obtained tomograms was 268 ± 34 nm (*n* = 17). The tilt-series were aligned using ETomo from the IMOD software package (University of Colorado, USA) (Kremer et al., 1995) by cross-correlation. The tomograms were reconstructed using the weighted-back projection algorithm in ETomo from IMOD. The image j software was used to determine particle size at the different time points of the developmental cycle.

### Sequencing, assembly, and genome annotation of *R. massiliensis*

Genomic DNA of *R. massiliensis* was sequenced by MiSeq Technology (Illumina, Inc., San Diego, CA) using the paired-end and mate-pair applications in parallel in a 2 × 251 bp run for each bar-coded library. The reads were assembled *de novo* into contigs using Mira 3.4 (Chevreux et al., 1999). SSPACE software v1.0 combined with GapFiller was used to enhance the assembly (Boetzer et al., 2011; Nadalin et al., 2012). Non-coding genes and miscellaneous features were predicted using RNAmmer (Lagesen et al., 2006), ARAGORN (Laslett and Canback, 2004), Rfam (Griffiths-Jones et al., 2003), PFAM (Punta et al., 2011), and Infernal (Nawrocki et al., 2009). Coding DNA sequences (CDSs) were predicted using Prodigal (Hyatt et al., 2010), and functional annotation was achieved using BLAST+ (Camacho et al., 2008) and HMMER3 (Eddy, 2011) against the UniProtKB (UniProt Consortium, 2011) database. Data for *R. massiliensis* were submitted to the EMBL database and was assigned Bio-projects number PRJEB6078; accession numbers for the genome at EMBL are CCSC01000001–CCSC01000005.

### Phylogenetic tree construction

The phylogenetic analyses were performed for the genes of *R. massiliensis* and the corresponding gene sequences available on the NCBI database. Multiple sequence alignments were performed using MUSCLE (Edgar, 2003) and curated using Gblocks (Talavera and Castresana, 2007). Phylogenetic trees were constructed using the PhyML Maximum Likelihood algorithm; the trees were visualized using MEGA v5 (Tamura et al., 2011).

## Results

### Culture and developmental characteristics

At 48 h after inoculation, it was possible to observe amoebae filled with small Gram-negative cocci, and the Gimenez staining assessed the intracellular nature of these small positive cocci. The bacteria appeared to be strict intracellular microorganisms unable to grow outside of the amoeba, because no growth could be detected in different media and under multiple atmospheric culture conditions in the absence of host amoeba. Among the tested temperatures ranging from 4 to 42°C, *R. massiliensis* grew only in amoeba at 28, 30, and 32°C, which corresponded to the *V. vermiformis* optimal growth temperature.

**Figure 1:**
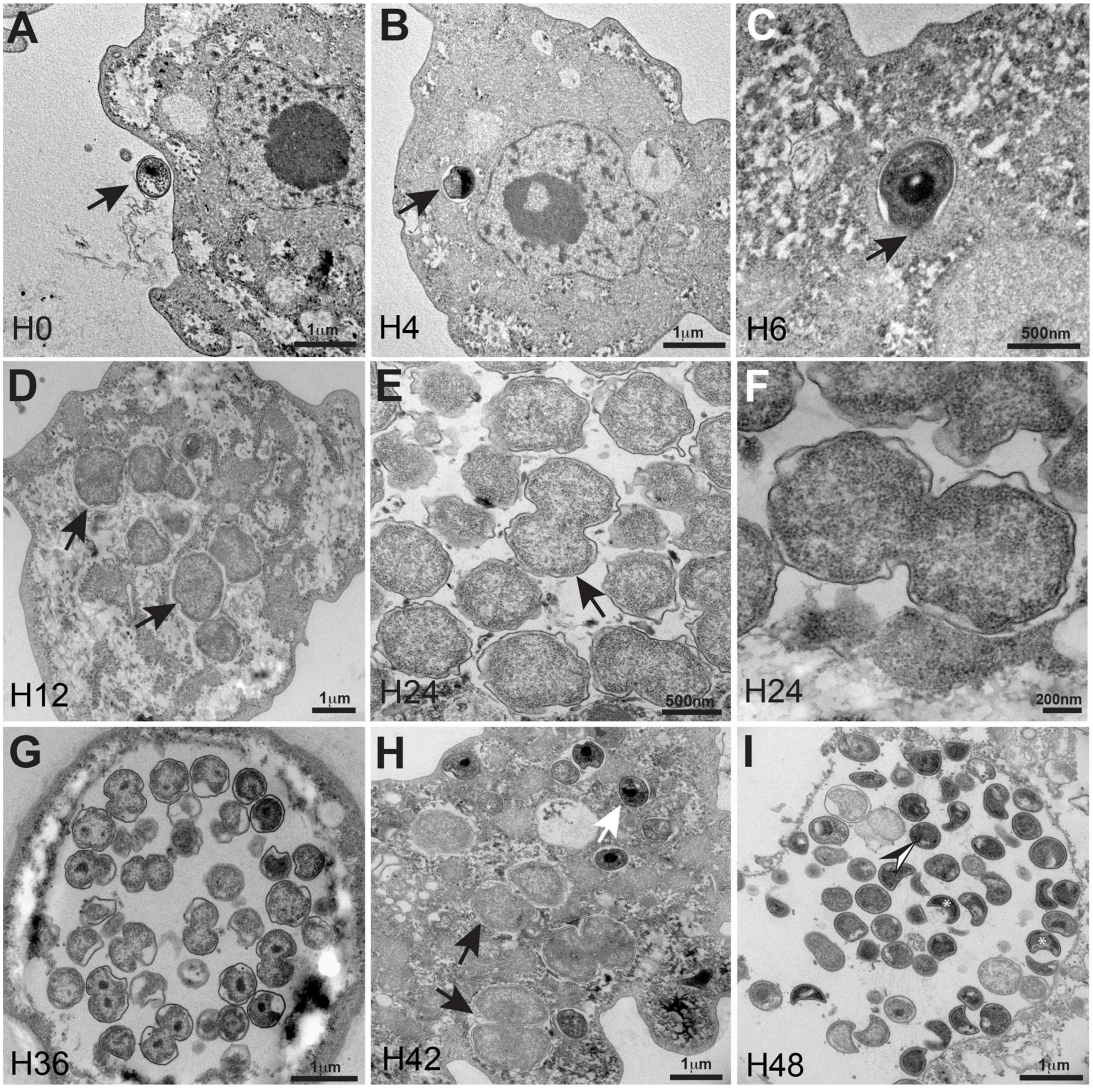
**Ultrastructural features of the ***R. massiliensis*** replication cycle in ***V. vermiformis*** (A)** Adhesion and phagocytosis of a *R. massiliensis* elementary body, by a trophozoite of *V. vermiformis* at 0 h p.i. **(B)** One EB already engulfed and internalized within the cytoplasm, seen near the nucleus. EB do not reside within vacuole (arrowhead). **(C)** Another typical EB can be seen within the cell host cytoplasm at 6 h p.i (arrowhead). In contrast to Rickettsia and other *Chlamydia* related to *Acanthamoeba*, no electron-translucent layers surrounding the intra-cellularly located bacteria could be observed. This is an evidence for the absence of inclusion vacuoles. **(D)** Arrowheads indicating constrictions of Reticulate bodies (RBs) undergoing their replicative stage where we observe an increase in size and a decrease in density. **(E)** Full replicative stage at 24 h p.i showing an increased number of *R. massiliensis* particles, hypodense RBs, at different stages of morphogenesis. **(F)** Higher magnification of the arrowed area in **(E)**, we can see the bacterium at the typical binary fission stage. (Tomographic reconstruction in Movie S1). **(G)** Completely infected *V. vermiformis* at 36 h p.i. After the growth and binary division, RBs reorganize, condensing to form infectious EBs. We note differentiation from hypodense to intermediate condensed particle and numerous bacteria scattered throughout the cytoplasm in various stages of differentiation. Condensed DNA is clearly visible in the two forming daughter cells. (Tomographic reconstruction in Movie S2). **(H)** Different stages of the *R. massiliensis* developmental cycle. RBs (black arrows), and EBs (white arrow) can be observed simultaneously within the cytoplasm of the *V. vermiformis* host cell, and do not reside within vacuoles. At 42 h p.i the co-presence of RBs and EBs signals the asynchronous cycle of *R. massiliensis*. **(I)** Ultrathin section of an infected amoeba, harboring the newly synthetized bacterial committee. The newly synthetized bacteria occupy the whole cell cytoplasm area. At 48 h p.i, we can see the different shapes of the newly differentiated, and highly condensed EBs but corresponding to only one rounded shape of the hyper dense EBs. (Tomographic reconstruction in Movie S3).

Electron microscopy of the bacterium revealed typical morphological characteristics of the *Chlamydiales* including the existence of a *Chlamydia*-like life cycle and indicated that this amoebal pathogen did not reside within a vacuole. The developmental cycle of *R. massiliensis* could be divided into two stages similar to the majority of *Chlamydiae*. Both the elementary bodies (EBs, the infectious non-replicative form that is hyperdense under electron microscopy) and the reticulate bodies (RBs, the replicative non-infectious form that is hypodense under electron microscopy) could be observed. The phagocytosis of EBs was the starting point of the cycle (Figure 1A). After phagocytosis and at time point H4 the bacteria were internalized within the cytoplasm. They could be seen near the host cell nucleus and were localized to a peri-nuclear location. No inclusion vacuoles were observed at these time points (arrowheads in Figures 1B,C). This process led to a phase that lasted from 6 to 8 h p.i. where no particles, more than the ones observed in the previous time points of infection at 4 h p.i, could be observed. Within 8–12 h following their internalization, the metabolically inert EBs underwent morphological changes and reorganized into the larger RBs. The bacteria began to lose their electron-density and progressively increased in size from coccoid forms 0.6 ± 0.2 μm in length to cocco-bacilli hyperdense forms 1.8 ± 0.4 μm in length and 1.6 ± 0.3 μm width. Between H8 and H12 the bacterial bodies continued to grow, forming large accumulations of amorphous material. These structures appeared to grow in the cytoplasm of the amoebae (Figure 1D). At 24 h p.i, the resulting RBs divided by binary fission; this stage lasted for the duration of the intracellular developmental cycle (Figures 1E,F and tomography reconstruction, Movie S1). After a period of growth and division, the RBs reorganized at ~36 h p.i., condensing to form infectious pre-mature EBs that would mature into the highly condensed EBs. (Figure 1G). Condensed DNA was clearly visible in the forming daughter cells (Figure 1G, tomography reconstruction, Movie S2). At 48 h p.i, the different shapes of the newly differentiated and highly condensed EBs were visible and almost filled amoeba cytoplasm (Figure 1I), but also induced amoeba burst and can be observed outside of a lysed amoeba The different shapes cited in the literature including crescent bodies (Rusconi et al., 2013) or transmission electron microscopy artifacts (Pilhofer et al., 2014) could be attributed to the rounded shape of the hyperdense EBs seen in different section cuttings (Figure 1I, and tomography reconstruction, Movie S3). These highly condensed EBs accumulated within the cytoplasm until the cell burst. Similar to the majority of *Chlamydiales* (Omsland et al., 2014), the developmental stage of C. *R. massiliensis* was somewhat asynchronous: the EBs began to accumulate in the cytoplasm at ~42 h p.i. while some RBs were still dividing (Figure 1H).

**Figure 2:**
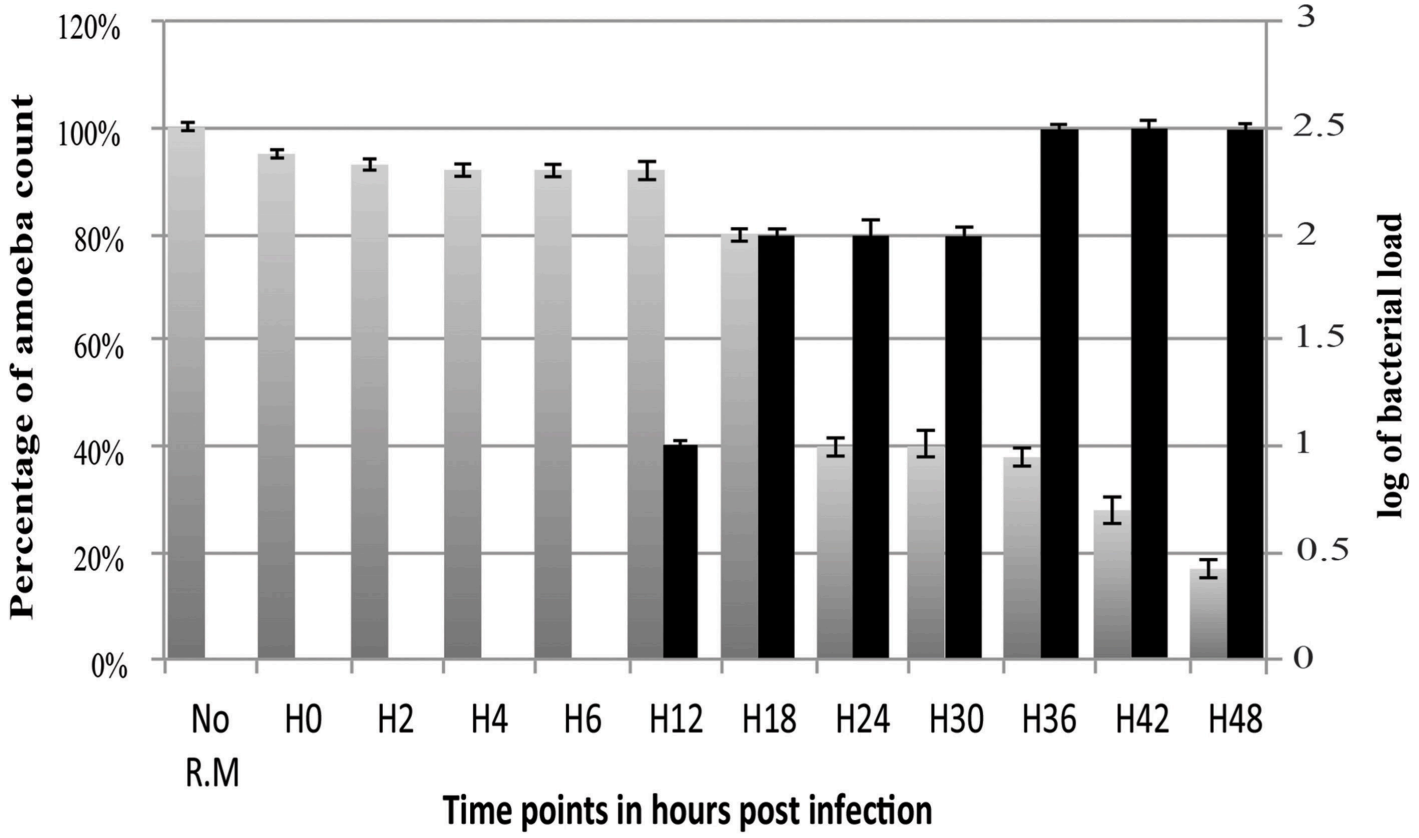
**Histogram of ***R. massiliensis*** cycle growth in ***V. vermiformis*** measured by real-time PCR**. X-axis corresponds to the cycle time points in hours (from 0 to 48 h). No R.M corresponds to the negative control, which is the non-infected amoeba). Y-axis to the right corresponds to the log of bacterial load (the log values are obtained after conversion of the Cycle threshold (Ct.) values based on standard curves realized with serial 1: 10 dilution starting with 10^7^ bacterial particles). Y-axis to the left corresponds to the percentage of amoeba concentrations quantified on kovaslides. This relative quantification by real-time PCR showed the increase in bacterial multiplication coming along with the decrease of the amoeba concentration. No bacterial DNA was detected from H0 till H6. Bacterial titers begin to be detected at H12 p.i, Higher titers are from H18 till H30 with a plateau from H36 until H48 p.i.

The amount of *R. massiliensis* DNA increased steadily to reach a plateau at H30. Another increase with a plateau was observed between H36 and H48 (Figure 2). This might reflect the re-infection of uninfected amoebae still present in the culture. Additionally, it could also be due to the non-synchronized infectious cycle of this bacterium as described above (Figure 1H). Infection with *R. massiliensis* led to a nearly complete lysis of the amoebae within 48 h. After 48 h in culture, the amount of infected amoebae decreased by ~85% compared to the non-infected culture of *V. vermiformis* used as a negative control. This latter culture showed no detectable loss of amoebae over the same experiment duration.

The *R. massiliensis* grew only in *V. vermiformis*. *A. castellanii* and *D. discoideum* seem to be completely protected from *R. massiliensis* infection or not permissive to the bacterial growth in comparison to *V. vermiformis*, where the culture and the relative quantification by real-time PCR showed the increase in bacterial multiplication in *V. vermiformis* at H24, but especially at H72, and H120 with an increase in the number of bacteria per milliliter of about 2 Log in 5 days. In parallel, no growth nor a cytopathic effect in *A. castellanii* and *D. discoideum* cells were detected. A small decrease of the bacterial inoculum used for the infection at H0 was still detectable at H120 in *A. castellanii* indicating that bacteria were still viable after this period. For *D. discoideum*, the bacterial load dramatically decreased, mentioning that the bacteria were digested or no more viable (Figure S1).

### The *R. massiliensis* genome

**Figure 3:**
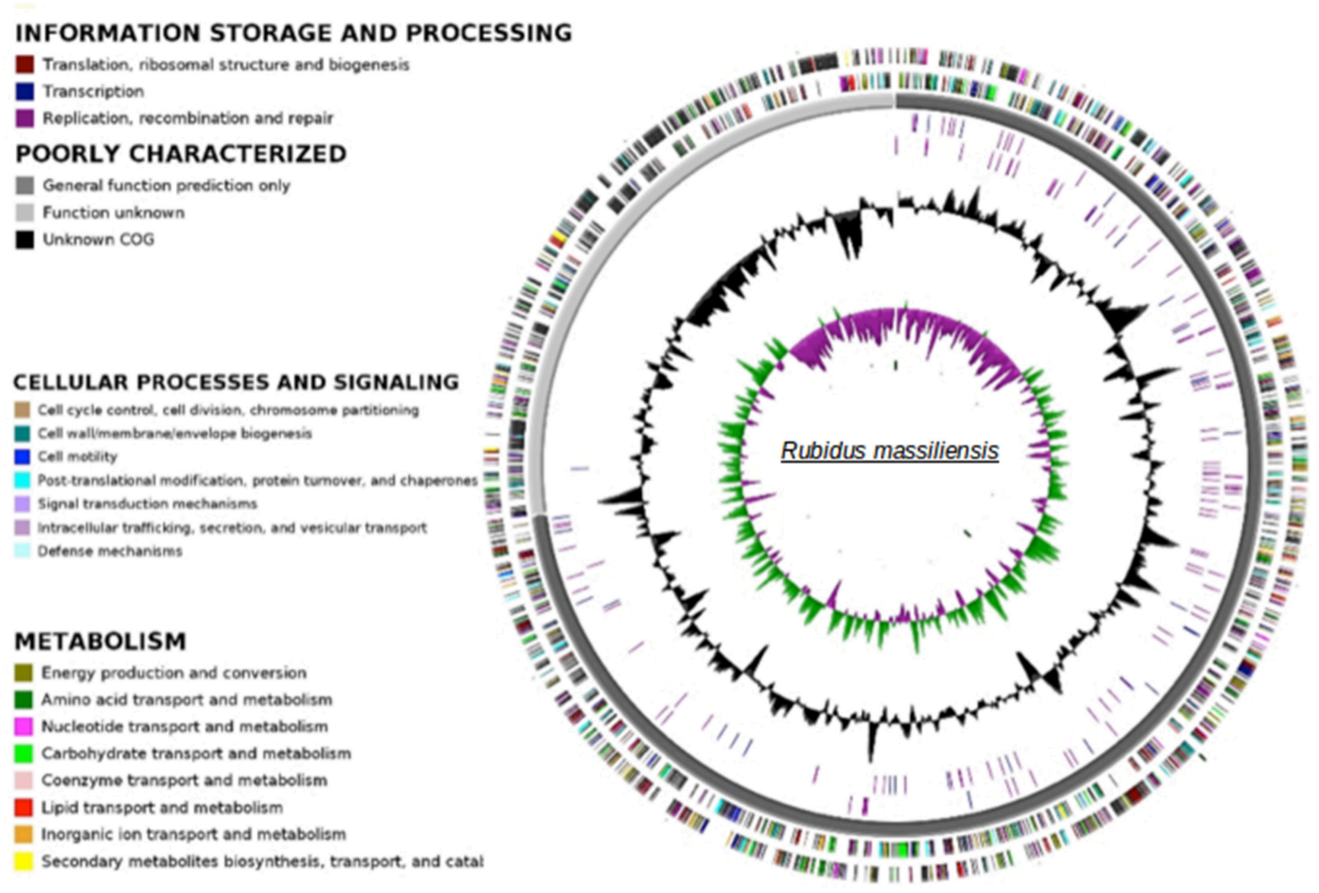
**Circular representation of the ***R. massiliensis*** chromosome**. Circles from the center to the outside: GC skew (green/purple), GC content (black), RNA on forward strand (tRNA in blue, rRNA in purple), RNA on reverse strand (tRNA in blue, rRNA in purple), scaffolds in alternative grays, genes on forward strand colored by COGs categories, genes on reverse strand colored by COGs.

The paired-end and mate-paired libraries allowed the generation of 1,087,468 and 1,321,408 reads respectively. The genome of *R. massiliensis* consists of a single chromosome assembled into three contigs (Figure 3) and two putative plasmids (Figure S2). The chromosome is an estimated 2.8 Mbp in size and has a GC content of 32.45%. In total, 2299 protein coding sequences (CDS) were identified in the chromosome sequence, as well as 5 rRNA and 36 tRNA genes. A huge excess of bacterial (96.47) over eukaryotic (3.04) homologs was observed; moreover, a total of 21.91% of the CDS had no orthologs with other *Chlamydiae* (Figure S3). Most of the bacterial homologs were amoebae parasites. Only eight best matches of all ORFs were shared with Archaea and one with an unclassified phage. ORFans (ORFs having no match in the NCBI bank) represented 17.26% (397) of the predicted chromosomes. According to the Pillonel et al. method (Pillonel et al., 2015), *R. massiliensis* was classed in the Parachlamydiaceae family based on the RNA 16S and 23S percent identity (Figure S4). Four proteins (Chromosomal replication initiation protein, 2-Oxoglutarate dehydrogenase subunit E1, hypothetical protein 325, and Enoyl-ACP reductase) were used to classify this bacterium as a new genus.

**Figure 4:**
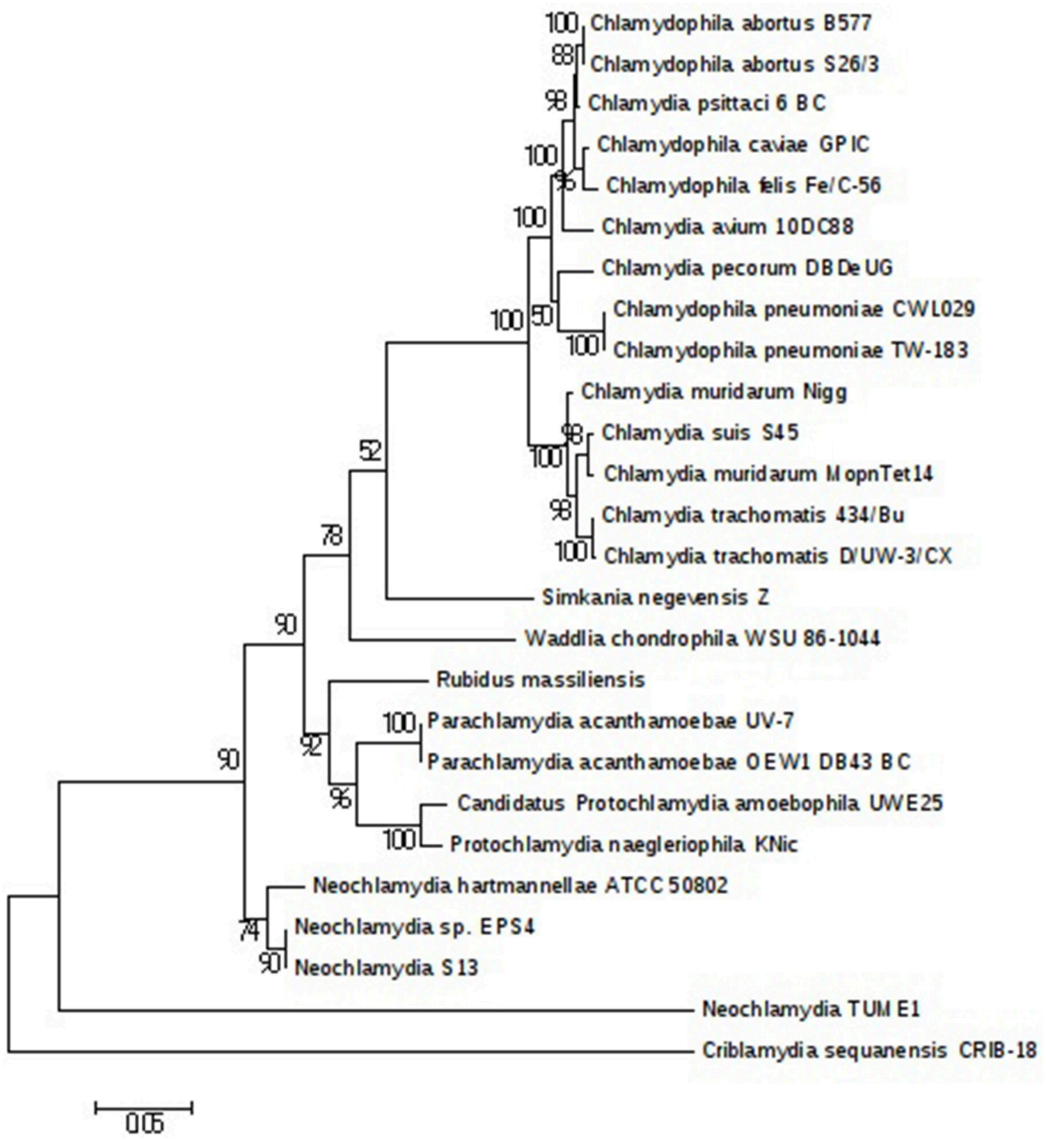
*****Chlamydiales*** members clustering according to a phylogenetic tree analysis**. Maximum-likelihood (PhyML) phylogenetic tree calculated with JTT+G substitution model With the RNA 16S sequences of 26 *Chlamydiales members*. Bootstrap Proportion values are indicates at the node.

The plasmids (named pRm1, pRm2) are 39,075 and 80,897 bp in size with GC contents of 29.85 and 40.18%, respectively. The average read depth of the *R. massiliensis* pRm1 (78.44x) and pRm2 (73.20x) compared to the chromosome (31.27x) could imply that the plasmids are present as ~2 to 3 copies per cell. pRm1 is circular and encodes for 40 proteins, 18 of which are homologs of the *Simkania negevensis* plasmid. We identified two virulence plasmid proteins and an addiction module toxin RelE that has not been found in other members of *Chlamydia*, which explains the preservation of the daughter cells' plasmids. pRm2 was classified as a plasmid based on its variation in GC content. Both two plasmids present an important reads coverage. The circular structure of pRm2 was verified by PCR primers (5′–3′: CCACATCCCAGGTGATATTGC ATGCCCTTGCTACAATTTACTG). pRm2 is predicted to contain 107 proteins, including four coding for antibiotic resistance and three for heavy metal resistance (two for copper and one for tellurite). pRm2 contains a zeta toxin that is thought to be part of a post-segregational killing (PSK) system involved in the killing of plasmid-free cells (Meinhart et al., 2003). A Fic/DOC protein and 20 phage proteins were also identified in this plasmid. The *Criblamydia sequanensis* plasmid 1 (strain CRIB-18) shares over 70% nucleotide identity with the *R. massiliensis* pRm2. Indeed, 48 ORFs of their genome repertoire were homologous (Figure S5). These two plasmids present similar or close GC content (40.18 and 40.8%, respectively) and are almost at the same size (80,897 bp and 89,525 bp, respectively). The phylogenetic tree of 16S rRNA sequences constructed for a representative set of the *Chlamydia* sequences available in the nr database clustered *R. massiliensis* with *Neochlamydia. hartmannellae* (Figure 4). These two sequences comprised a clade that clustered with *Parachlamydiaceae*. The bootstrap value relating *R. massiliensis* to the *Parachlamydiaceae* was 73%.

### The cell division machinery and genomic signatures of an intracellular pathogen

To further investigate the specific cell division mechanism of *R. massiliensis*, we searched for genes implicated in the cell division of seven *Chlamydiales* (Table S1) using a BLASTp search against a house database of proteins involved in cell division. The comparison of *R. massiliensis* to the other *Chlamydiales* shows a lack of some genes, such as *fts*A, *fts*K, and *yof* A. The lack of a number of genes involved in cell division (i.e., *fts*Z and *fts*B) was observed in all seven studied *Chlamydiales* (Greub, 2010; Bavoil et al., 2013). *R. massiliensis* unlike other *Chlamydia*-like organisms has not the *ftsK* gene, which was also lacked in the *Chlamydiaceae* members. Additionally, *R. massiliensis*, as the *Protochlamydia amoebophila* and *Chlamydiaceae*, is lacking of the HTH type transcriptional regulator YofA. However, *R. massiliensis* like *Protochlamydia acanthamoebae*, has more genes in their repertoire than the members of *Chlamydia*.

**Figure 5:**
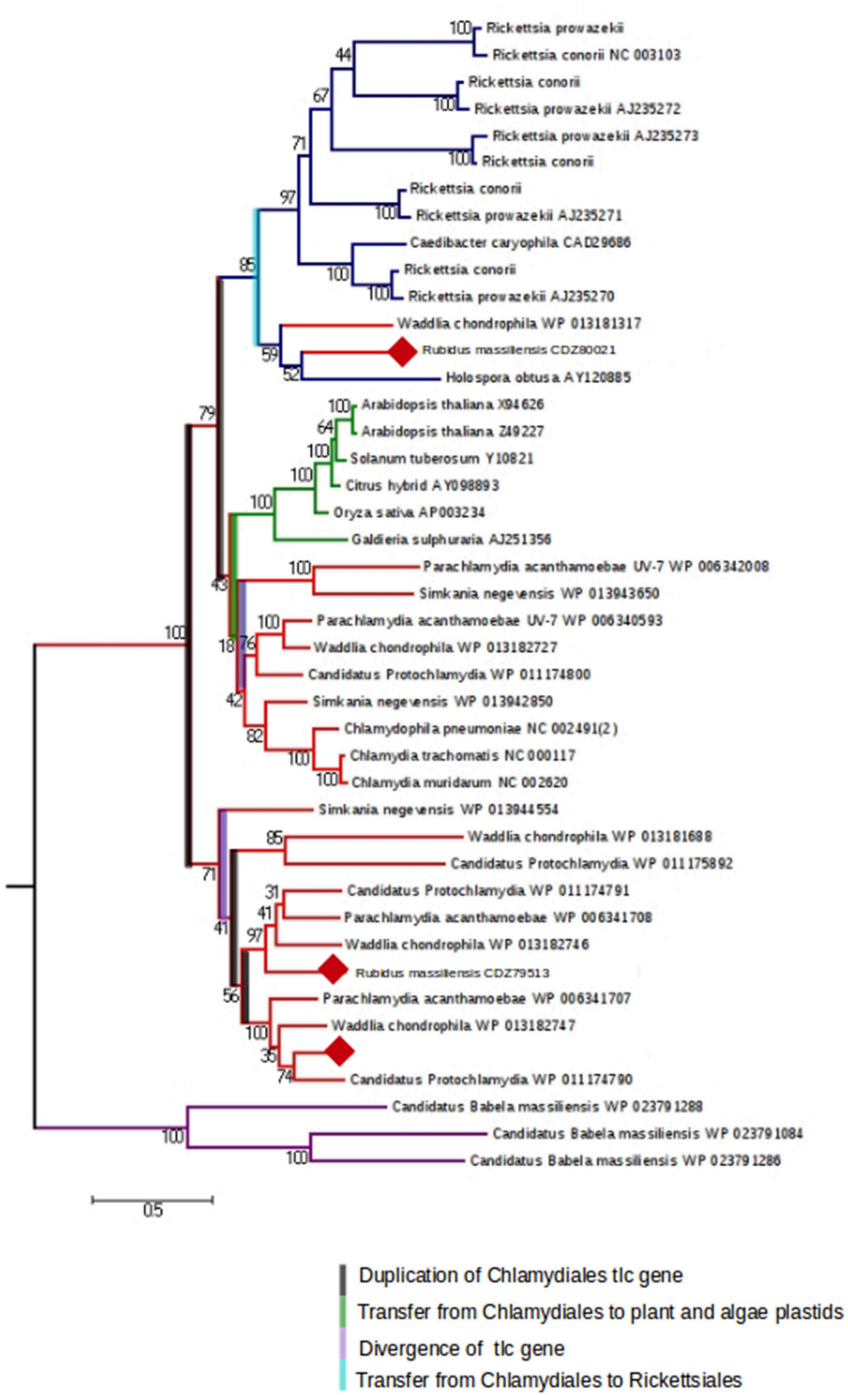
**The maximum-likelihood (PhyML) phylogenetic tree inferred from amino acid sequences of the ADP/ATP translocase of ***Parachlamydia acanthamoebae*** UV-7, ***Candidatus, Protochlamydia***, ***Waddlia chondrophila***, ***Simkania negevensis***, ***R. massiliensis***, ***Chlamydiaceae***, Rickettsiales, and plant and algal plastids**. Bootstrap proportion values are indicated at the node.

We were interested then in studying the energy input of these bacteria. The ADP/ATP translocase exchanges bacterial ADP for ATP and allows energy parasitism. Three translocases (BN1013_00005, BN1013_00006, and BN1013_00525) similar to those of *Waddlia chondrophila* and *Protochlamydia amoebophila* were identified in the *R. massiliensis* genome. A phylogenetic tree of ADP/ATP translocase amino acid sequences built for a representative set of bacterial sequences (*P. acanthamoebae UV-7, Protochlamydia amoebophila, W. chondrophila, S. negevensis, R. massiliensis, Chlamydiaceae, Rickettsiales*, and plant and algal plastids), available in the NR database was generated based on a maximum-likelihood (PhyML) (Figure 5). The *tlc* gene of *C. chondrophil* and *R. massiliensis* clustered with the *Rickettsiales*. The Chlamydiales members were clustered with the Rickettsiales by a node having a strong bootstrap value of 85%. The topology of the tree suggests that the *tlc* gene was transferred to plant and algal plastids before the transfer to *Rickettsiales*. Some *Chlamydia*-like organisms samples contained several ATP/ADP translocases. Hence, we hypothesize that the presence of several duplications explains the presence of multiple copies. Indeed, the Figure 5 shows four duplications of the *tlc* gene. 31 genes encoding the major structural components of the secretion apparatus and chaperones of The Type III secretion system (T3SS) were identified and are arranged in 13 genetic loci. Over half of these genes were regrouped in three genetic loci (Figure S6) and share a conserved synteny with the other *Chlamydiales* members. Duplication of some genes was observed in *R. massiliensis* and *W. chondrophila*. Deletion and rearrangement were observed in *S. negevensis and Protochlamydia amoeabophila*.

## Discussion

An emerging interest in the protozoa-associated *Chlamydiales* has allowed progress in genome sequencing and analysis and an expansion in the number of known *Chlamydiales*. Recently, five members of the *Chlamydiales* order were discovered and their genomes sequenced, increasing the number of the *Chlamydiales* families to seven: *Chlamydiaceae, Candidatus* Parilichlamydiaceae, *Criblamydiaceae, Rhabdochlamydiaceae, Simkaniaceae, Parachlamydiaceae*, and *Waddliaceae* (Greub, 2010; Bavoil et al., 2013).

Electron microscopy examination of *R. massiliensis* revealed a typical *Chlamydiales* morphology with a *Chlamydia*-like life cycle (Amann et al., 1997). However, in contrast to other *Chlamydia* species, it did not reside within a vacuole implying a complete absence of the process of inclusion vacuoles already described for *Chlamydiales* in *Acanthamoeba* (Michel et al., 1994; Amann et al., 1997). The absence of the process of inclusion vacuoles has already been observed in the *Vermamoeba* previously named *Hartmanella* (Horn et al., 2000; Michel et al., 2004). Our findings suggest that this developmental cycle without vacuoles could be linked to this particular specie of *Chlamydiae* or perhaps to its *Vermamoeba* host. Indeed the fact that a similar finding was previously described with an endoparasite of *Vermamoeba* (Horn et al., 2000) and the specificity of *R. massiliensis* to its host left the question unanswered. The isolation of other *Chlamydiae* capable of infecting both *Vermamoeba* and other types of amoebae but also a comparative genome analysis of *R. massiliensis* and the endoparasit of Vermamoeba could address this question. This capability is may be due to the capability of *R. massiliensis* to degrade the vacuole via its phospholipase activity. Both the genomes of *Criblamydia* and *R. massiliensis* contained homologs of this phospholipase (CSEC-1990 and BN1013-00614).

*R. massiliensis* shares a high sequence similarity with other *Chlamydiae*. More than 60% of the best hits from the Blastp results correspond to other *Chlamydiales* proteins, and the presence of two plasmids is a feature unique to this group of *Chlamydia*. The pRm1 presented a GC content lower than the genome, which was in agreement with the findings of Moran et al. (Moran, 2002). In contrast, pRm2 presented a GC content that was only 10% higher than the genome (Nishida, 2012). *R. massiliensis* presented the second longest plasmid after that of *C. sequanensis*. As suggested by Bertelli et al. (2014), the analysis of the two mega-plasmid sequences will increase our understanding of the evolution of the *Chlamydiales* plasmids. Three *tlc* gene sequences were identified in the *R. massiliensis* genome. Therefore, we constructed a phylogenetic tree based on the model of Greub et al. (Greub and Raoult, 2003) that included a large number of *Chlamydia tlc* genes. Using this enriched tree, some contradictions and new findings were observed (Greub and Raoult, 2003; Schmitz-Esser et al., 2004). The *tlc* genes of two *Chlamydiales* were clustered with those of *Rickettsiales*, indicating that this gene was a copy of the ancestral *tlc* gene that was transmitted to the *Rickettsiales*. Furthermore, several duplications were observed, attesting to the complexity of this gene's evolutionary history. In conclusion, the discovery of new Chlamydiae infecting protozoa such as *R. massiliensis* may be useful tools to elucidate *Chlamydiales* evolution and obligate intracellular parasitism.

### Short description of “*R. massiliensis*”

*R. massiliensis* (Ru.bi'dus, pertaining to the ruby, the precious gemstone that was the aspect of this bacterium the first time we observed it under negative staining electron microscopy; mas.si.li.en′sis, L. fem. adj. *massiliensis*, referring to Massilia, Latin name of Marseille, where the strain was characterized). Phylogenetic position, Chlamydiales phylum; Gram-negative; mature infectious particles have coccus shaped morphology 0.6 ± 0.2 μm in size; accession numbers for the genome at EMBL are CCSC01000001–CCSC01000005; not cultivated on cell-free media; obligate intracellular pathogen of *V. vermiformis; two* morphologies typical of *Chlamydiales* according to stage in the developmental cycle; multiplication through binary fission.

## Author contributions

JB performed cultivation, TEM, and wrote the paper, SB did bioinformatic analysis and wrote the paper, JB performed TEM, OC, did bioinformatic analysis, CB did genome sequencing, IP performed isolation and culture, DR corrected the manuscript, BL concepted the study and corrected the manuscript.

## Funding

SB was supported by the Fondation Mediterranee Infection.

### Conflict of interest statement

The authors declare that the research was conducted in the absence of any commercial or financial relationships that could be construed as a potential conflict of interest.

## Supplementary material

Movies are shown in order and linked to the IHU (méditerranée infection) web site: http://www.mediterranee-infection.com/article.php?laref=344&titre=rubidus-massiliensis.

Figure S1 **Host range: Histogram of ***R. massiliensis*** growth or replication in three types of amoeba: ***V. vermiformis, A. Castellanii***, and ***D. discoideum***, measured by real-time PCR to test ***R. massiliensis*** host range for 5 days post infection**. Data are the means SD from three independent experiments performed in triplicate. (H0, H24, H72, and H120 correspond to the different time points in hours). [Vv No R.M, *V. Vermiformis* negative control (not infected); Vv R.M, *V. vermiformis* infected with *R. massiliensis;* AC No R.M, *A. Castellanii* negative control (not infected); AC R.M, *A. Castellanii* infected with *R. massiliensis;* Dd No R.M, *D. discoideum* negative control (not infected); Dd R.M, *D. discoideum* infected with *R. massiliensis*]. Y-axis corresponds to the log of bacterial load (the log values are obtained after conversion of the Cycle threshold (Ct.) values based on standard curves realized with serial 1:10 dilution starting with 10^7^ bacterial particles).

Figure S2 **Circular representation of the ***R. massiliensis*** plasmids**. Circles from the center to the outside: GC skew (green/purple), GC content (black). Genes on forward strand colored by COGs categories, CDS on forward strand colored in blue, genes on reverse strand colored by COGs, CDS on reverse strand colored in blue.

Figure S3 **Best hits repartition of the Blastp results**. We note a huge excess of Bacterial (96.47) over Eukaryotic (3.04). 21.91% of the CDS, had no orthologs with other *Chlamydiae*. Among the bacterial homologs, most are amoeba parasites (Rickettsia, legionella…etc.).

Figure S4 *****Rubidus massiliensis*** classification scheme based on both 16S–23S rRNA gene sequences and four proteins**. Classification based on the percentage of sequence identity of nine protein sequences (sucA, 2-Oxoglutarate dehydrogenase subunit E; fabI, Enoyl-ACP reductase; dnaA, Chromosomal replication initiation protein; hyp325, Hypothetical protein) of the *R. massiliensis* and all other sequenced members of the order *Chlamydiales*.

Figure S5 **Comparative analysis of ***Chlamydiales*** plasmids. (A)**: Alignement of *R. massiliensis* pRm2 plamsid and the *C. sequanencis* plasmid1, the ORFs having homology were linked by a blue line. **(B)**: Venn diagramm showing the genomics comparison of four *Chlamydiae* plamsids.

Figure S6 **Comparison of the synteny of three genetic loci of ***R. massiliensis, P acanthamoebae, Protochlamydia amoebophila, W. chondrophila, S. negevensis, C. trachomatis*** 434 and ***C pneumoniae*** AR39**. T3SS genes were represented by colored arrows (the arrows of the structural genes were circled by a green line and those of Chaperones by a red line). Genes having an hypothetical function were represented by white arrows and those having a function by hatched arrows. Coordinate of the genetic loci on the chromosome **(A)**: 351168–353891, **(B)**: 1329268–1332657, and **(C)**: 1537631–1550426.

Table S1 **Distribution of cell division genes among 7 *Chlamydiales* members**.

Movie S1 **Tomographic reconstruction, and a three-dimensional segmentation of a dividing ***R. massiliensis***' particle, in an infected amoeba at 36 h post-infection**. The acceleration voltage was 200 kV, the magnification was 25,300, and the pixel size was 0.434 nm. The tilt series ranged from –45° to +55° in 1° steps. The three-dimensional 3D reconstruction generated from the corresponding tilt series was 238 nm thick (176.30 nm shown in the movie). The bacterial cytoplasm does not present DNA condensation, thereby the bacteria was referred as hypo-dense. The segmented 3D model of the most-external bacteria plasma membrane is colored green. The 3D model highlights the middle tightening of the dividing bacteria. No cytoplasmic bacterial internal membrane was visible along the tomogram thickness.

Movie S2 **Tomographic reconstruction of a dividing ***R. massiliensis***' particle in an infected amoeba at 36 h post-infection**. The acceleration voltage was 200 kV, the magnification was 29,000, and the pixel size was 0.364 nm. The tilt series ranged from –45° to +55° in 2° steps. The three-dimensional 3D reconstruction generated from the corresponding tilt series was 236 nm thick. Condensed DNA was clearly visible in the two forming daughter cells, thereby the bacteria was referred as hyper-dense. No cytoplasmic bacterial internal membrane was visible.

Movie S3 **Tomographic reconstruction of hyper-dense ***R***. massiliensis particles in an infected amoeba at 36 h post-infection**. The acceleration voltage was 200 kV, the magnification was 9600, and the pixel size was 1.09 nm. The tilt series ranged from –50° to +55° in 1° steps. The three-dimensional 3D reconstruction generated from the corresponding tilt series was 277 nm thick. *R. massiliensis* hyper-dense particles presented multiple morphologies. The white arrow points to a single particle presenting a crescent-shape in the top of the tomogram and a circular (reticulate)–shape in the bottom of the tomogram.
