# Supplementary figures and images for: Developmental Cycle and Genome Analysis of “Rubidus massiliensis,” a New Vermamoeba vermiformis Pathogen
Source: Front Cell Infect Microbiol. 2016 Mar 15;6:31. doi: 10.3389/fcimb.2016.00031 (PMC4791399; doi:10.3389/fcimb.2016.00031)

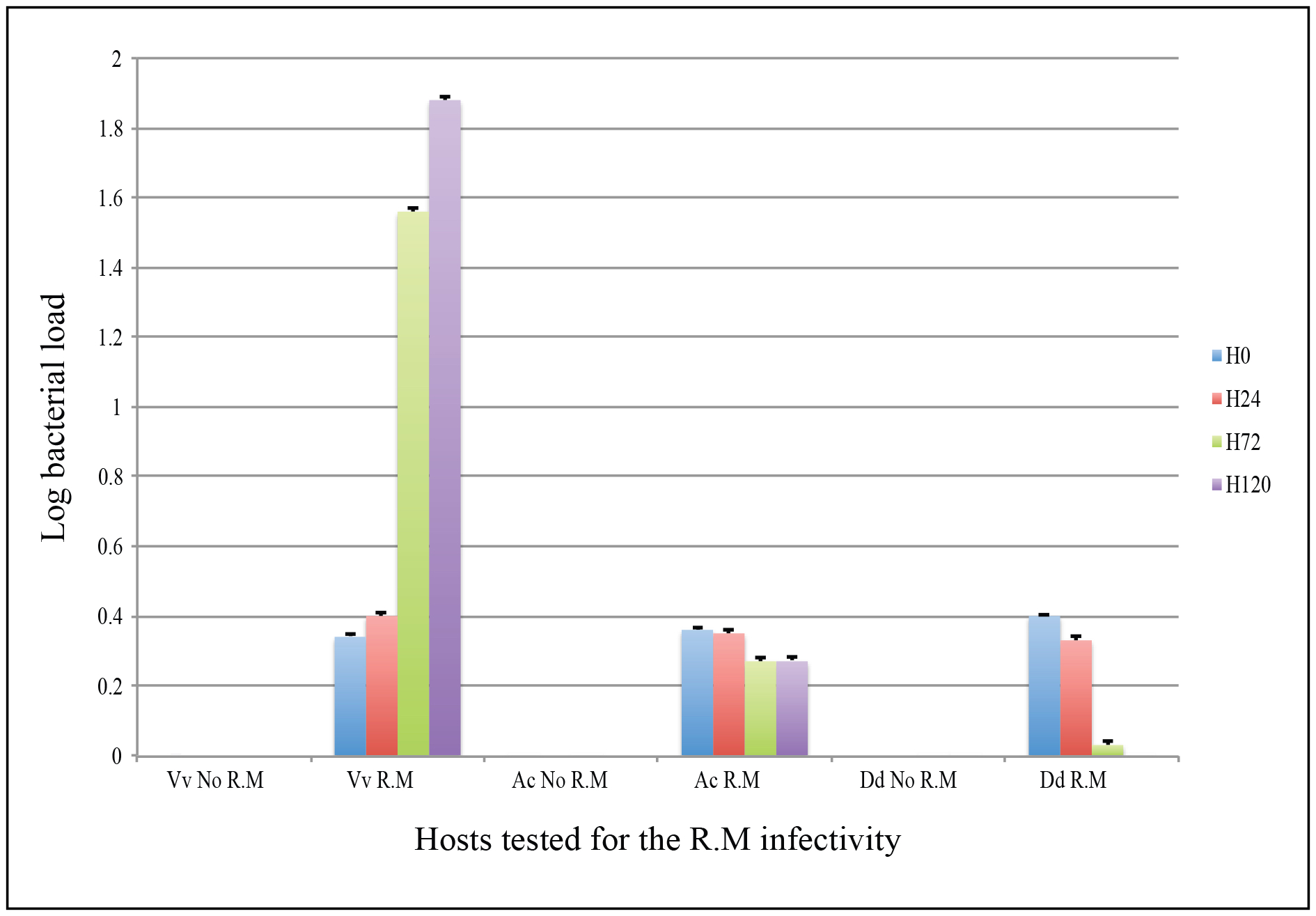

Supplement: Figure S1 — Host range: Histogram of R. massiliensis growth or replication in three types of amoeba: V. vermiformis, A. Castellanii, and D. discoideum, measured by real-time PCR to test R. massiliensis host range for 5 days post infection. Data are the means SD from three independent experiments performed in triplicate. (H0, H24, H72, and H120 correspond to the different time points in hours). [Vv No R.M, V. Vermiformis negative control (not infected); Vv R.M, V. vermiformis infected with R. massiliensis; AC No R.M, A. Castellanii negative control (not infected); AC R.M, A. Castellanii infected with R. massiliensis; Dd No R.M, D. discoideum negative control (not infected); Dd R.M, D. discoideum infected with R. massiliensis]. Y-axis corresponds to the log of bacterial load (the log values are obtained after conversion of the Cycle threshold (Ct.) values based on standard curves realized with serial 1:10 dilution starting with 107 bacterial particles). [file Image1.JPEG]

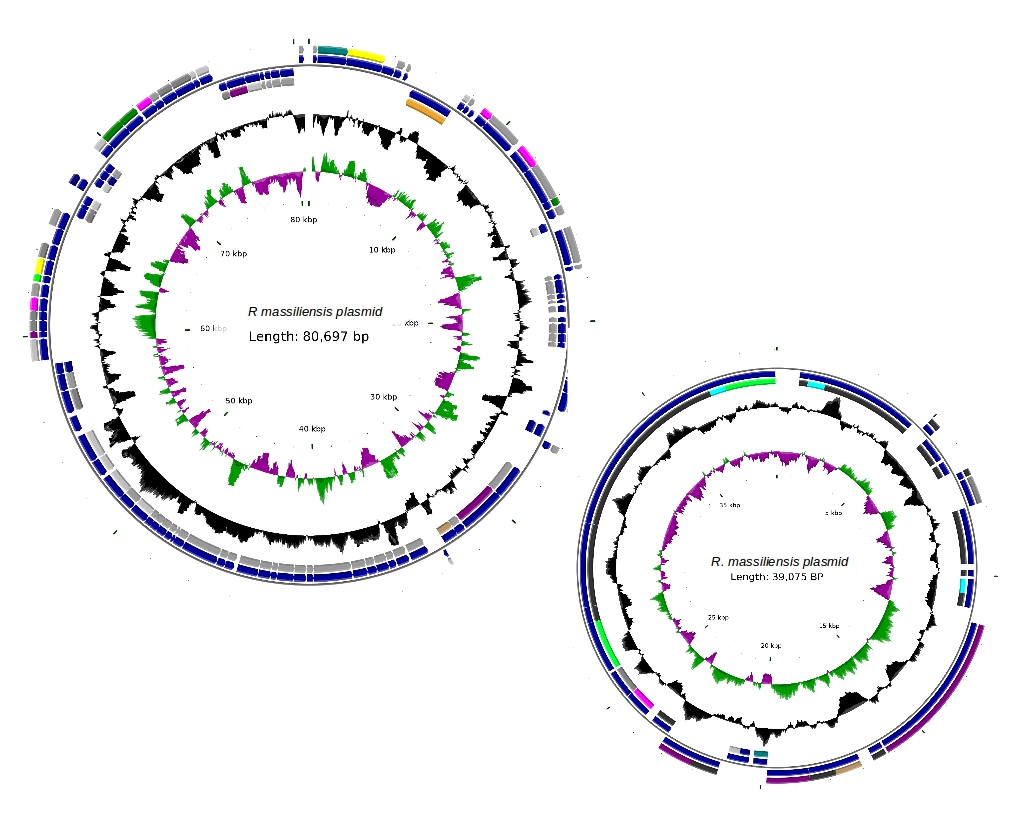

Supplement: Figure S2 — Circular representation of the R. massiliensis plasmids. Circles from the center to the outside: GC skew (green/purple), GC content (black). Genes on forward strand colored by COGs categories, CDS on forward strand colored in blue, genes on reverse strand colored by COGs, CDS on reverse strand colored in blue. [file Image2.JPEG]

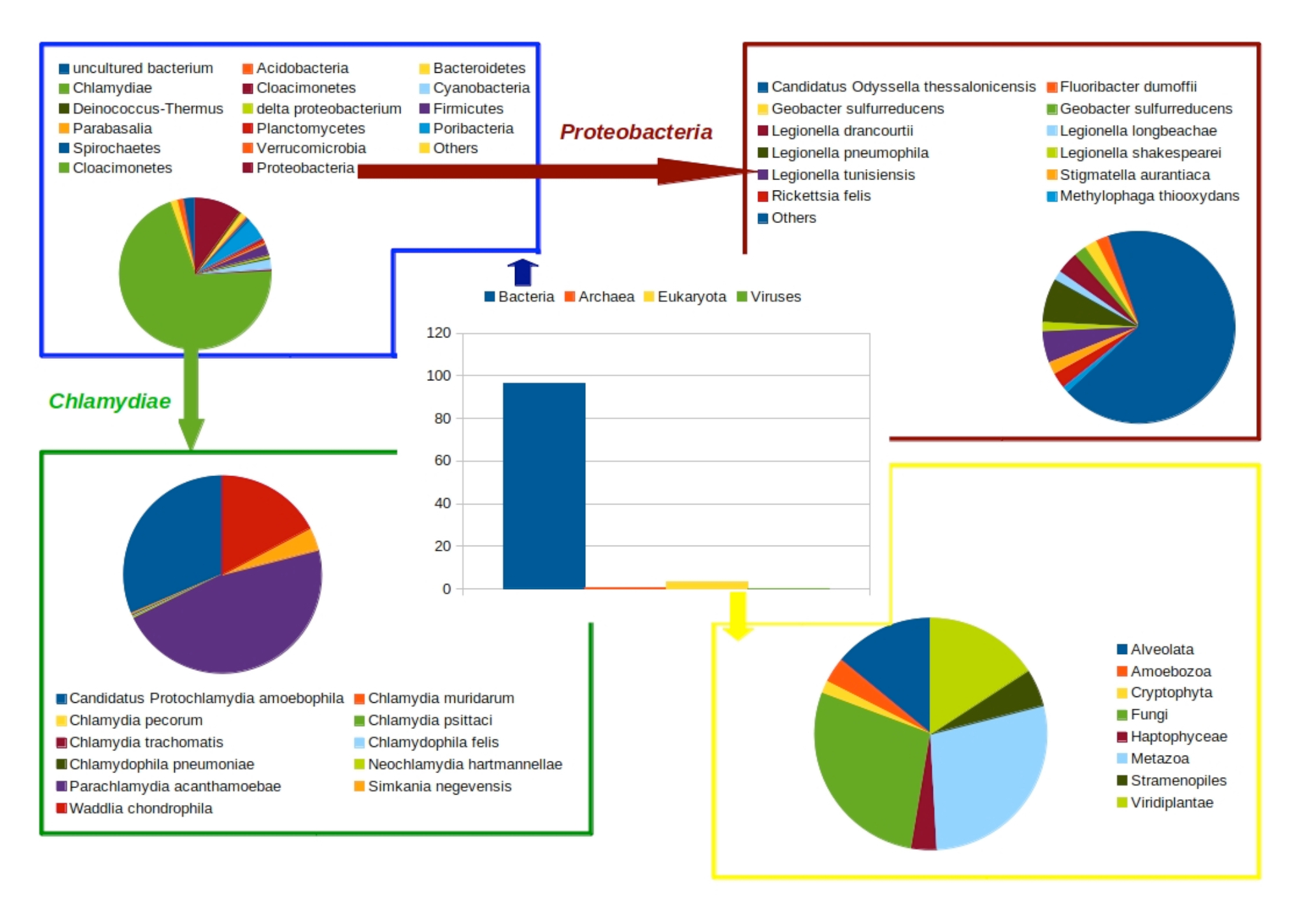

Supplement: Figure S3 — Best hits repartition of the Blastp results. We note a huge excess of Bacterial (96.47) over Eukaryotic (3.04). 21.91% of the CDS, had no orthologs with other Chlamydiae. Among the bacterial homologs, most are amoeba parasites (Rickettsia, legionella…etc.). [file Image3.JPEG]

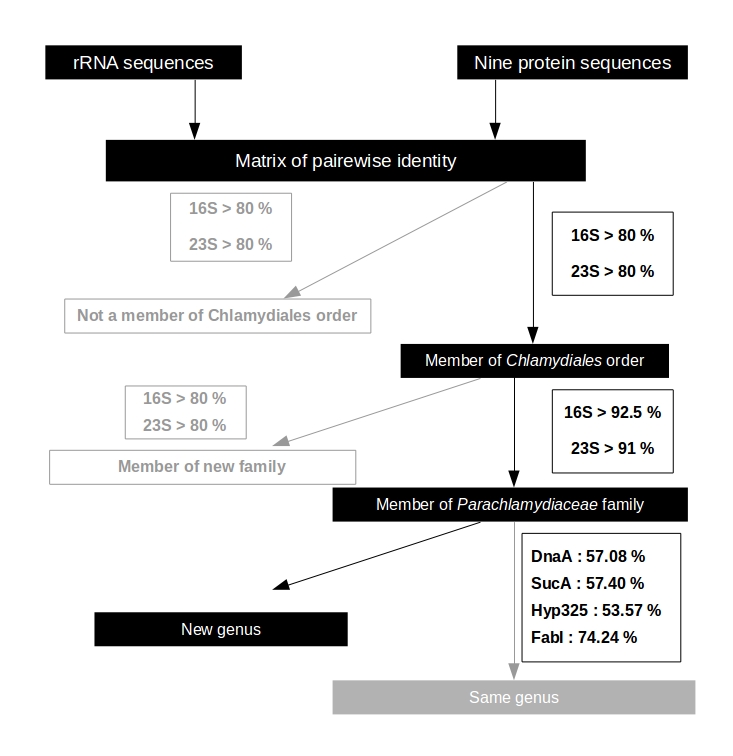

Supplement: Figure S4 — Rubidus massiliensis classification scheme based on both 16S–23S rRNA gene sequences and four proteins. Classification based on the percentage of sequence identity of nine protein sequences (sucA, 2-Oxoglutarate dehydrogenase subunit E; fabI, Enoyl-ACP reductase; dnaA, Chromosomal replication initiation protein; hyp325, Hypothetical protein) of the R. massiliensis and all other sequenced members of the order Chlamydiales. [file Image4.JPEG]

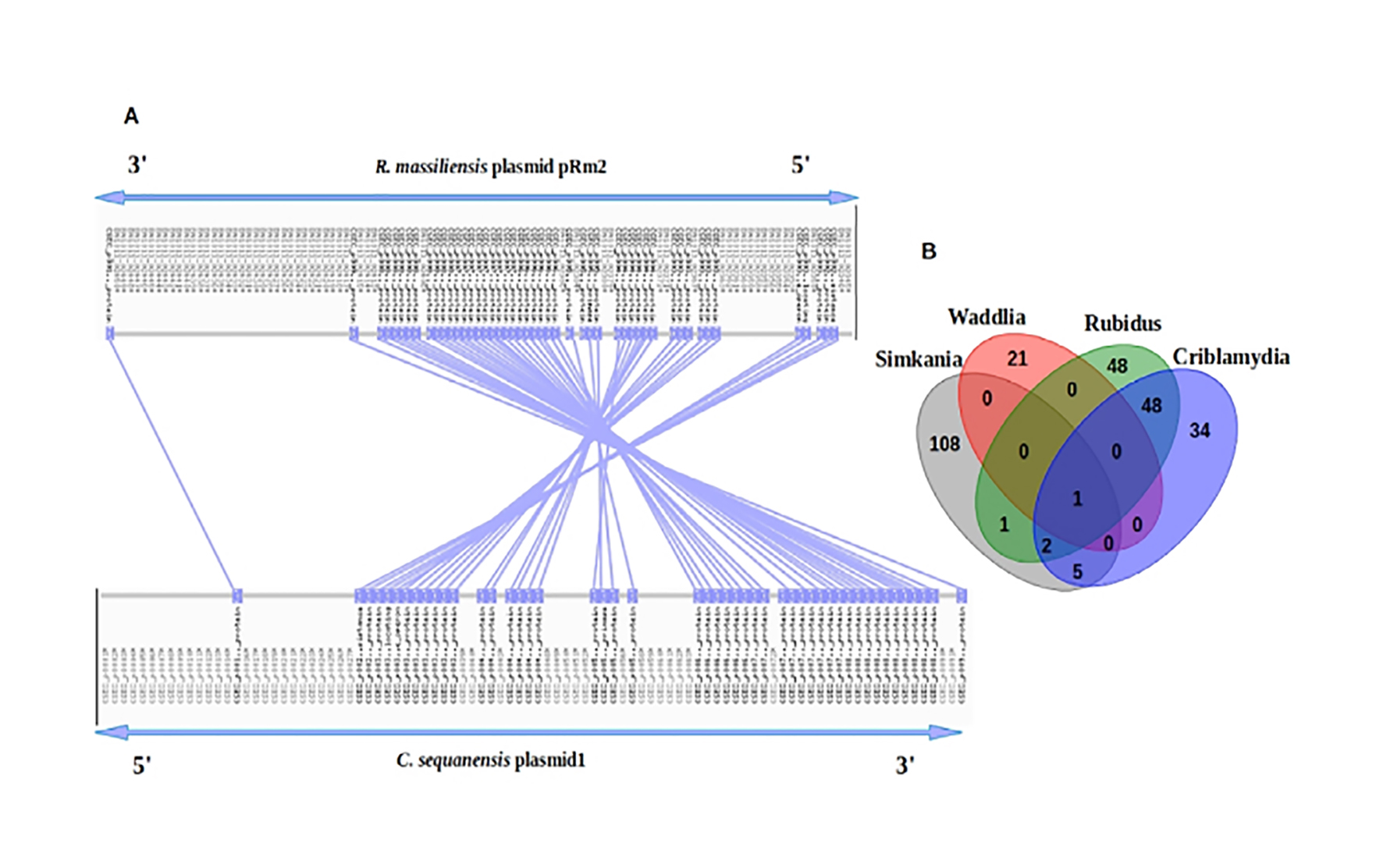

Supplement: Figure S5 — Comparative analysis of Chlamydiales plasmids. (A): Alignement of R. massiliensis pRm2 plamsid and the C. sequanencis plasmid1, the ORFs having homology were linked by a blue line. (B): Venn diagramm showing the genomics comparison of four Chlamydiae plamsids. [file Image5.JPEG]

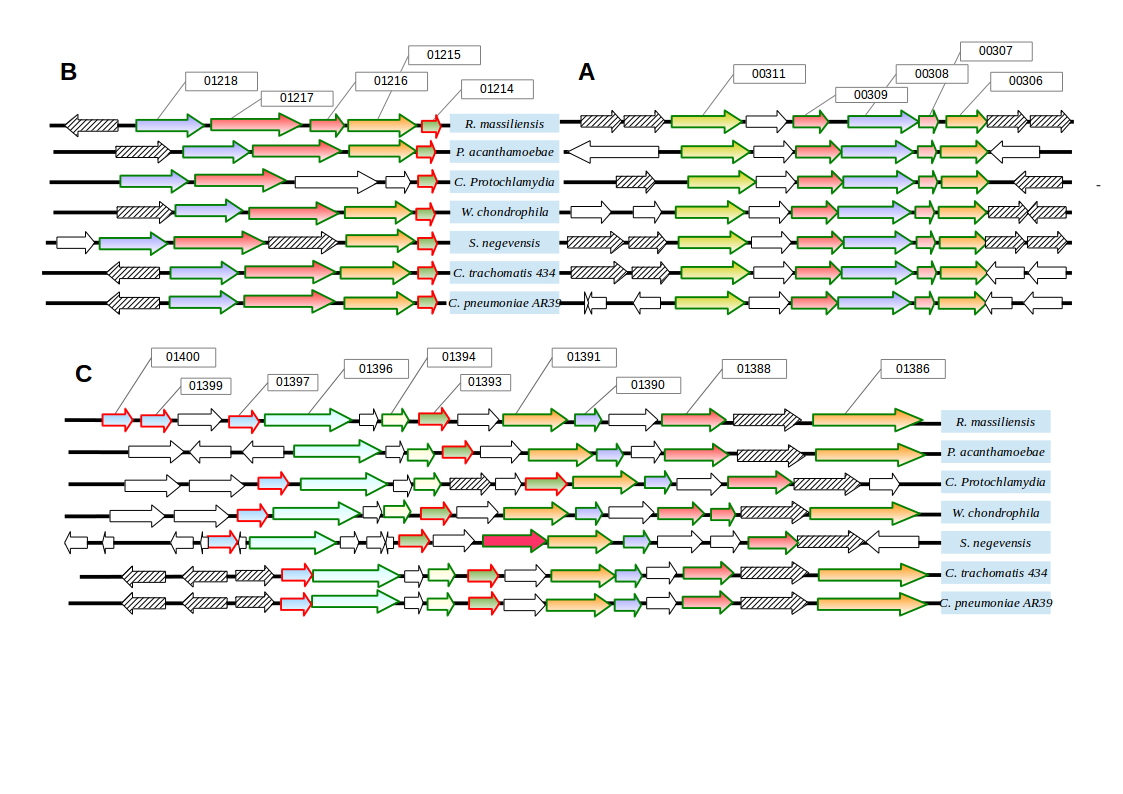

Supplement: Figure S6 — Comparison of the synteny of three genetic loci of R. massiliensis, P acanthamoebae, Protochlamydia amoebophila, W. chondrophila, S. negevensis, C. trachomatis 434 and C pneumoniae AR39. T3SS genes were represented by colored arrows (the arrows of the structural genes were circled by a green line and those of Chaperones by a red line). Genes having an hypothetical function were represented by white arrows and those having a function by hatched arrows. Coordinate of the genetic loci on the chromosome (A): 351168–353891, (B): 1329268–1332657, and (C): 1537631–1550426. [file Image6.JPEG]
