# Supplementary material for: Developmental Cycle and Genome Analysis of “Rubidus massiliensis,” a New Vermamoeba vermiformis Pathogen
Source: Front Cell Infect Microbiol. 2016 Mar 15;6:31. doi: 10.3389/fcimb.2016.00031 (PMC4791399; doi:10.3389/fcimb.2016.00031)
Supplement: Movie S1 — Tomographic reconstruction, and a three-dimensional segmentation of a dividing R. massiliensis' particle, in an infected amoeba at 36 h post-infection. The acceleration voltage was 200 kV, the magnification was 25,300, and the pixel size was 0.434 nm. The tilt series ranged from –45° to +55° in 1° steps. The three-dimensional 3D reconstruction generated from the corresponding tilt series was 238 nm thick (176.30 nm shown in the movie). The bacterial cytoplasm does not present DNA condensation, thereby the bacteria was referred as hypo-dense. The segmented 3D model of the most-external bacteria plasma membrane is colored green. The 3D model highlights the middle tightening of the dividing bacteria. No cytoplasmic bacterial internal membrane was visible along the tomogram thickness. [file DataSheet1.docx]

**Supplementary information legends**

**SI Table S1**: Distribution of cell division genes among 7 *Chlamydiales* members

Please check the submitted excel file: Table S1

**SI Figure S1:** **Host range: Histogram of *R. massiliensis* growth or replication in three types of amoeba:**

*V. vermiformis, A. Castellanii,* and *D. discoideum*, measured by real-time PCR to test *R. massiliensis* host range for 5 days post infection. Data are the means SD from three independent experiments performed in triplicate. (H0, H24, H72, and H120 correspond to the different time points in hours). (Vv No R.M = *V. Vermiformis* negative control (not infected), Vv R.M = *V. vermiformis* infected with *R. massiliensis*, AC No R.M = *A. Castellanii* negative control (not infected), AC R.M = *A. Castellanii* infected with *R. massiliensis*, Dd No R.M = *D. discoideum* negative control (not infected), Dd R.M = *D. discoideum* infected with *R. massiliensis*). Y-axis corresponds to the log of bacterial load (the log values are obtained after conversion of the Cycle threshold (Ct.) values based on standard curves realised with serial 1:10 dilution starting with 10^7^ bacterial particles).

**SI Figure S2**: Circular representation of the *R. massiliensis* plasmids. Circles from the center to the outside: GC skew (green/purple), GC content (black). Genes on forward strand colored by COGs categories, CDS on forward strand colored in blue, genes on reverse strand colored by COGs, CDS on reverse strand colored in blue.

**SI Figure S3**: Best hits repartition of the Blastp results. We note a huge excess of Bacterial (96.47) over Eukaryotic (3.04). 21.91% of the CDS, had no orthologs with other *Chlamydiae*. Among the bacterial homologs, most are amoeba parasites (Rickettsia, legionella...etc).

**SI Figure S4: *Rubidus massiliensis* classification scheme based on both 16S–23S rRNA gene sequences and four proteins.** Classification based on the percentage of sequence identity of nine protein sequences (sucA: 2-Oxoglutarate dehydrogenase subunit E, fabI: Enoyl-ACP reductase, dnaA: Chromosomal replication initiation protein, hyp325: Hypothetical protein) of the *Rubidus massiliensis* and all other sequenced members of the order *Chlamydiales*.

**SI Figure S5: Comparative analysis of *Chlamydiales* plasmids. A**: Alignement of *R. massiliensis* pRm2 plamsid and the *C. sequanencis* plasmid1, the ORFs having homology were linked by a blue line. **B:** Venn diagramm showing the genomics comparison of four *Chlamydiae* plamsids.

**SI Figure S6:** Comparison of the synteny of three genetic loci of *R. massiliensis*, *P acanthamoebae*, *Protochlamydia amoebophila*, *W. chondrophila*, *S. negevensis*, *C. trachomatis* 434 and *C pneumoniae* AR39. T3SS genes were represented by colored arrows (the arrows of the structural genes were circled by a green line and those of Chaperones by a red line). Genes having an hypothetical function were represented by white arrows and those having a function by hatched arrows. Coordinate of the genetic loci on the chromosome (A) :  351168..353891, (B) : 1329268..1332657 and (C): 1537631..1550426.

**Movies captions**

**SI Movie 1. Tomographic reconstruction, and a three-dimensional segmentation of a dividing *R. massiliensis’* particle, in an infected amoeba at 36 hours post-infection.**

The acceleration voltage was 200 kV, the magnification was 25,300, and the pixel size was 0.434 nm. The tilt series ranged from -45° to +55° in 1° steps. The three-dimensional 3D reconstruction generated from the corresponding tilt series was 238 nm thick (176.30 nm shown in the movie). The bacterial cytoplasm does not present DNA condensation, thereby the bacteria was referred as hypo-dense. The segmented 3D model of the most-external bacteria plasma membrane is colored green. The 3D model highlights the middle tightening of the dividing bacteria. No cytoplasmic bacterial internal membrane was visible along the tomogram thickness.

**SI Movie 2. Tomographic reconstruction of a dividing *R. massiliensis’* particle in an infected amoeba at 36 hours post-infection**.

The acceleration voltage was 200 kV, the magnification was 29,000, and the pixel size was 0.364 nm. The tilt series ranged from -45° to +55° in 2° steps. The three-dimensional 3D reconstruction generated from the corresponding tilt series was 236 nm thick. Condensed DNA was clearly visible in the two forming daughter cells, thereby the bacteria was referred as hyper-dense. No cytoplasmic bacterial internal membrane was visible.

**SI Movie 3. Tomographic reconstruction of hyper-dense *R.* massiliensis particles in an infected amoeba at 36 hours post-infection.**

The acceleration voltage was 200 kV, the magnification was 9,600, and the pixel size was 1.09 nm. The tilt series ranged from -50° to +55° in 1° steps. The three-dimensional 3D reconstruction generated from the corresponding tilt series was 277 nm thick. *R. massiliensis* hyper-dense particles presented multiple morphologies. The white arrow points to a single particle presenting a crescent-shape in the top of the tomogram and a circular (reticulate) -shape in the bottom of the tomogram.

Movies are shown in order and linked to the IHU (méditerranée infection) web site:

<http://www.mediterranee-infection.com/article.php?laref=344&titre=rubidus-massiliensis>
